# Prefrontal Transcranial Direct Current Stimulation Globally Improves Learning but Does Not Selectively Potentiate the Benefits of Targeted Memory Reactivation on Awake Memory Consolidation

**DOI:** 10.3390/brainsci11081104

**Published:** 2021-08-21

**Authors:** Médhi Gilson, Michael A. Nitsche, Philippe Peigneux

**Affiliations:** 1 UR2NF—Neuropsychology and Functional Neuroimaging Research Unit, affiliated at CRCN, Centre for Research in Cognition and Neurosciences, Avenue F.D. Roosevelt 50, 1050 Bruxelles, Belgium; medhi.gilson@hotmail.com; 2 UNI—ULB Neuroscience Institute, Université Libre de Bruxelles (ULB), avenue F.D. Roosevelt 50, 1050 Bruxelles, Belgium; 3 Department of Psychology and Neuroscience, Leibniz Research Center for Working Environment and Human Factors (IfADo), 44139 Dortmund, Germany; nitsche@ifado.de; 4 Department of Neurology, University Medical Hospital Bergmannsheil, 44789 Bochum, Germany

**Keywords:** memory, emotion, targeted memory reactivation, transcranial direct current stimulation

## Abstract

Targeted memory reactivation (TMR) and transcranial direct current stimulation (tDCS) can enhance memory consolidation. It is currently unknown whether TMR reinforced by simultaneous tDCS has superior efficacy. In this study, we investigated the complementary effect of TMR and bilateral tDCS on the consolidation of emotionally neutral and negative declarative memories. Participants learned neutral and negative word pairs. Each word pair was presented with an emotionally compatible sound. Following learning, participants spent a 20 min retention interval awake under four possible conditions: (1) TMR alone (i.e., replay of 50% of the associated sounds), (2) TMR combined with anodal stimulation of the left DLPFC, (3) TMR combined with anodal stimulation of the right DLPFC and (4) TMR with sham tDCS. Results evidenced selective memory enhancement for the replayed stimuli in the TMR-only and TMR-sham conditions, which confirms a specific effect of TMR on memory. However, memory was enhanced at higher levels for all learned items (irrespective of TMR) in the TMR-anodal right and TMR-anodal left tDCS conditions, suggesting that the beneficial effects of tDCS overshadow the specific effects of TMR. Emotionally negative memories were not modulated by tDCS hemispheric polarity. We conclude that electrical stimulation of the DLPFC during the post-learning period globally benefits memory consolidation but does not potentiate the specific benefits of TMR.

## 1. Introduction

Memory consolidation is the process by which novel and fragile memory traces are progressively transformed into more robust representations [1]. Consolidation is possibly supported by the offline (i.e., after actual practice in the learning phase) replay of the neuronal activity that subtends learning processes [2]. Supporting this hypothesis, animal and human studies evidenced the reinstatement of learning-related brain activity during offline post-training periods, both when awake [3,4,5,6] and during sleep [7,8,9,10,11,12]. Several studies have reported better consolidation in declarative memory after sleep than wakefulness, which is mostly associated with the triggering of learning-related brain activity during slow wave sleep (SWS; for reviews see, e.g., [13,14,15,16]), others showed that quiet post-learning wakefulness can also benefit memory consolidation (e.g., [4,5,6,17,18,19,20,21]). For instance, memory for stories was found to be superior after 10 min spent in a wakeful resting state (i.e., the participant being not actively involved in any task) than after an equivalent period of time spent in active wakefulness (e.g., playing an unrelated game) [18]. To some extent, the neural oscillatory mechanisms involved in memory formation while awake might share similarities with sleep [22]. Accordingly, Tambini and colleagues [20] found that enhanced hippocampal-cortical coordination during wakeful rest after learning is predictive of memory performance. Furthermore, increased slow oscillatory activity (1 Hz) and reduced alpha (8–12 Hz) activity during quiet rest awake periods were associated with memory improvement for learned stories, suggesting that slow oscillations while awake might promote the dialogue between hippocampal and cortical regions [23], like during sleep [2]. Therefore, post-training resting wakefulness might improve memory performance not only by reducing ongoing interferences, but also by providing a neural milieu that favors the interactions between subcortical hippocampal areas and cortical regions subtending memory consolidation, including prefrontal areas [5].

Targeted memory reactivation (TMR), i.e., the presentation of learning-related cues during offline post-learning periods, can additionally enhance memory consolidation (for reviews see, e.g., [16,24]). For instance, TMR during post-training wakefulness was found to rescue targeted memories from forgetting [25,26], although this was not always replicated (see, e.g., [27,28] for a lack of effect on vocabulary learning). TMR was also found detrimental to putting memories in a labile state and increasing sensitivity to interference [29], and in some cases was more [26] or solely [30,31] beneficial during sleep (for a review see, e.g., [32]).

Another promising technique to promote learning and memory consolidation processes is transcranial direct current stimulation (tDCS), which modulates cortical excitability [33,34,35,36]. Anodal excitatory stimulation of the left dorsolateral prefrontal cortex (DLPFC) during encoding improves declarative memory [37,38], whereas cathodal inhibitory stimulation exerts a detrimental effect [38,39]. Similarly, cathodal stimulation of the left DLPFC impairs recognition performance, whereas it tends to be improved by anodal stimulation. Transcranial DCS was also found to be effective on associative memory, either using a single 20 min anodal tDCS session [40,41] or theta frequency-modulated oscillatory anodal tDCS [42] over the left posterior parietal cortex before learning. Besides pre-learning effects, tDCS during actual encoding is also beneficial for memory. For instance, anodal tDCS over the left prefrontal cortex during learning was shown to improve episodic memory in older adults [43]. Similarly, Leach et al. [44] evidenced a benefit of anodal stimulation of the left dorsolateral prefrontal cortex (DLPFC) using a face-name associative memory task, but in young adults only. Furthermore, tDCS may also benefit offline consolidation mechanisms. Indeed, although 20 min of anodal stimulation over the temporoparietal cortex did not modify the learning rate of object locations in elderly participants, it enhanced delayed free recall performance as measured one week later [45]. Similarly, anodal stimulation over the premotor cortex during rapid eye movement sleep [46] or while awake after learning [47] was shown to benefit the consolidation of a motor sequence. Additionally, tDCS over the left DLPFC after encoding is beneficial for verbal episodic memory in older adults [43]. Taken together, these studies suggest that tDCS can improve episodic memory formation and consolidation. It must be taken into account that a recent meta-analysis indicates that the effects of tDCS on episodic memory are moderated by stimulation parameters; more specifically stimulation duration and the choice of the memory task moderate anodal tDCS effects [48].

Emotional and arousing memories are usually better remembered than memories without any affective load (for a review see [49]). A potential hemispheric lateralization in the processing of emotion has been proposed. According to the right hemisphere hypothesis, the right hemisphere would be involved in the general processing of emotions [50]. In contrast, the valence specific hypothesis posits that the left hemisphere is dominant for positive emotions, and the right hemisphere for negative ones [51,52].

To the best of our knowledge, it is currently unknown whether TMR reinforced by simultaneous tDCS during the offline wake period has superior efficacy for the consolidation of verbal declarative memories. In the present study, we tested this hypothesis using TMR alone or in combination with tDCS over the dorsolateral prefrontal cortex (DLPFC) after the learning of pairs of words. Additionally, we investigated the effect of tDCS lateralization on the consolidation of emotionally neutral and negative word pairs, considering a potential hemispheric lateralization in the processing of emotions [51,53]. Based on the right hemisphere and the valence specific hypotheses, we expected the combination of excitatory anodal stimulation over the right DLPFC and inhibitory cathodal stimulation over the left DLPFC to benefit the consolidation of negative memories more so than tDCS with reversed polarity (left anodal/right cathodal).

In the present study, healthy young participants learned a list of neutral and negative word pairs. Each word pair was associated with an emotionally compatible sound at learning. Participants then spent 20 min awake in a quiet environment in one of four possible stimulation conditions, i.e., TMR-only (half of the sounds associated with the word pairs to remember replayed during the 20 min consolidation interval), TMR-anodal left tDCS (TMR plus anodal stimulation on left DLPFC and cathodal stimulation on right DLPFC), TMR-anodal right tDCS (identical with reversed polarity), or TMR-sham tDCS (TMR plus tDCS for 15 s only at the beginning of the 20 min period). We predicted better memory performance for cued word pairs than for non-cued word pairs, and that this effect would be potentiated by tDCS. Additionally, we expected a specific modulation of negative memories in the TMR-anodal right tDCS condition.

## 2. Materials and Methods

### 2.1. Participants

Seventy-two healthy participants gave their written informed consent to participate in this study approved by the Faculty Ethics committee at the Université Libre de Bruxelles (ULB). Three of them were excluded because they did not participate in the entirety of the experiment. The sixty-nine remaining participants were native French speakers, right-handed, free of medication known to influence sleep quality and/or mood. They reported not suffering or having suffered from neurological or psychiatric disorders. Participants were randomly assigned to one of four possible conditions (see explanations below): TMR-only (*n* = 16; Male 43.8%), TMR-anodal left tDCS (*n* = 16; Male 37.5%), TMR-anodal right tDCS condition (*n* = 17; Male 35.3%), or TMR-sham tDCS condition (*n* = 20; Male 45%). An a priori power analysis (G*Power 3, [54]) indicated that for an average cueing medium effect size = 0.50 (based on reported small to moderate TMR effects, e.g., [16]), at least 16 subjects per group (condition) are needed in the context of a mixed-design ANOVA with four repeated measures ((cued vs. not cued) × (neutral vs. negative) words) and four different between-subject conditions (TMR-only, TMR-anodal left tDCS, TMR-anodal right tDCS, TMR-sham tDCS), considering a power of 0.95.

Pre-study examination evidenced alexithymia scores below the cut-off score (mean ± standard deviation 41.08 ± 9.2, cut-off score 61; Alexithymia Toronto Scale, [55]) and vocabulary knowledge within normative values (score range 25–40/44; Mill-Hill Vocabulary Scale, [56]). Age (mean per group range 22.4–23.3 years; *p* = 0.82), alexithymia scores (mean per group range 37.7–43.6; *p* = 0.24) and vocabulary knowledge (mean per group range 31–32.5; *p* = 0.68) did not differ between conditions.

### 2.2. Material

A neutral and a negative list of unrelated French word pairs (18 pairs/list) were selected based on their emotional valence (neutral = 16.19 ± 13.7 vs. negative = 77.67 ± 19.8, t(1, 70) = 15.33, *p* < 0.0001; [57]). The two lists were equated according to lexical frequency (neutral = 54.06 ± 67.5 vs. negative = 56.14 ± 86.2, t(1, 70) = 0.11, *p* = 0.91; [58]), imaging valence (neutral = 5.43 ± 1.6 vs. negative = 4.85 ± 1.5, t(1, 70) = −1.55, *p* = 0.12; [59]), number of syllables (neutral = 1.75 ± 0.7 vs. negative = 1.97 ± 0.7, t(1, 70) = 1.32, *p* = 0.19) and number of letters (neutral = 5.86 ± 1.5 vs. negative = 6.38 ± 1.8, t(1, 70) = 1.34, *p* = 0.19).

Each word pair was randomly associated with a specific sound (duration 6 s), taken from the International Affective Digitized Sounds database (IADS, Bradley and Lang, 2007). Neutral word pairs were matched with neutral sounds and negative word pairs were matched with negative sounds. Negative sounds were selected based on high arousal and low pleasantness ratings on a 9-point Likert scale [60], while neutral sounds were selected according to middle arousal and middle pleasantness ratings (mean IADS pleasure rating neutral = 4.94 ± 0.21 vs. negative = 2.12 ± 0.38, t(1, 34) = 26.92, *p* < 0.001; mean IADS arousal rating neutral = 4.93 ± 0.8 vs. negative = 7.34 ± 0.5, t(1, 34) = −10.48, *p* < 0.001).

### 2.3. Procedure

**Figure 1:**
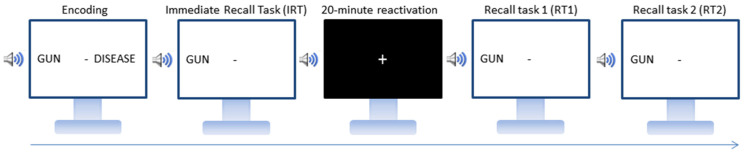
Experimental design. Participants learned word pairs associated with emotionally compatible sounds until reaching 75% correct recall in the immediate cued recall task (IRT). During the 20 min reactivation period in a wakeful resting state, the sounds associated with half of the neutral and negative word pairs were replayed three times, each alone (TMR-only) or concurrently with excitatory tDCS on the left (TMR-anodal left tDCS) or right (TMR-anodal right tDCS) hemisphere or in a sham tDCS condition (TMR-sham). Memory for all learned word pairs was then tested in a cued recall task immediately after (RT1) and one week later (RT2).

The experimental procedure is illustrated in Figure 1. All participants started with the encoding phase, during which each of the 36 word pairs was displayed one by one on a computer screen for 6 s while the associated sound was delivered through headphones. Between each word pair, a yellow fixation cross was displayed for 4 s. It turned red 1 s before the apparition of the next pair. The 36 word pairs were presented twice in random order. Participants were then administered an immediate cued recall test (IRT): the first word of each pair was displayed on the screen together with its associated sound, and participants had to type in the associated word. The correct response was then displayed on screen to promote error-free learning. If the participant’s total recall score was below the minimum criterion of 75% of correct responses, the incorrectly recalled word pairs were re-presented and then the IRT administered again on all pairs, until participants’ recall performance was at least 75%. After this learning session, participants were seated in a comfortable chair and asked to rest quietly for 20 min (wakeful resting period) while listening to a series of sounds. They were pseudo-randomly assigned to one of four possible conditions. In the TMR-only condition (*n* = 16 participants), half of the sounds (nine neutral and nine negative) associated with the learned word pairs were delivered three times each for a total of 54 auditory stimulations. The duration of each sound was 6 s, and the inter-stimulus interval randomly ranged between 6 and 12 s. In the TMR-anodal left tDCS condition, participants (*n* = 17) were administered the TMR protocol and received in parallel tDCS with the anode over the left DLPFC and the cathode over the right DLPFC. TMR-anodal right tDCS condition (*n* = 20) was identical to TMR-anodal left tDCS, except that the polarity of the electrodes was reversed (right anode/left cathode on tDCS). In the TMR-sham tDCS condition (*n* = 16), tDCS was delivered only for 15 s at the beginning of the TMR procedure.

Immediately after the 20 min stimulation period, participants were administered a cued recall task (RT1) on all word pairs presented in the learning session. RT1 was identical to the immediate recall task (IRT), except that participants did not receive any feedback on the correctness of their responses and there was no cut-off score. One week later, participants were administered a second cued recall session (RT2) in identical conditions and at the same time of the day as RT1.

Subjective sleepiness and objective vigilance were assessed at the beginning of the learning (KSS 1, PVT 1), RT1 (KSS 2, PVT 2) and RT2 (KSS 3, PVT 3) sessions using the Karolinska Sleepiness Scale (KSS, [61]) and the 10 min version of the Psychomotor Vigilance Task (PVT, [62]), respectively.

### 2.4. Transcranial Direct Current Stimulation

We chose here a bilateral dorsolateral prefrontal electrode montage as is used in several cognitive and clinical studies (e.g., for an overview see [36,63]). Conceptually, bilateral prefrontal stimulation should enhance the effects of stimulation under each electrode because of transcallosal connectivity, i.e., excitability-enhancing anodal tDCS will reduce the activity of the contralateral prefrontal cortex further because of enhanced transcallosal inhibition, and cathodal stimulation will have antagonistic effects on the contralateral prefrontal cortex. Thus, a kind of lateralized boosting effect can be assumed with this electrode arrangement. Noticeably, we chose not to position the return electrode on the supraorbital region close to the nearby ventral prefrontal areas, which are involved in emotion processing, and thus might have had an impact on cognitive performance on its own, which would have made interpretation of the results difficult and ambiguous with respect to localization of the effects.

**Figure 2:**
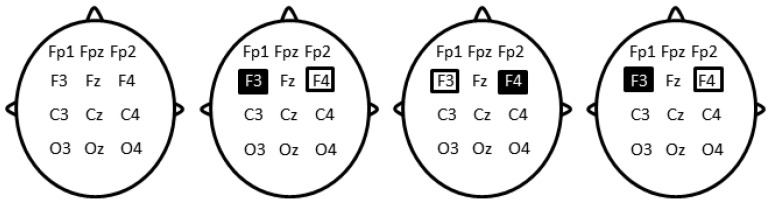
Schematic representation of electrodes’ position in the four conditions: (Left) TMR-only, no electrode positioned. (Left-middle) TMR-anodal left tDCS, anodal stimulation on the left DLPFC and cathodal stimulation on the right DLPFC for 20 min. (Right-middle) TMR-anodal right tDCS, cathodal stimulation of the left DLPFC and anodal stimulation of the right DLPFC for 20 min. (Right) Sham condition, anodal stimulation of the left DLPFC and cathodal stimulation of the right DLPFC for 15 s. The dark rectangle depicts the anode and the light rectangle depicts the cathode.

A pair of saline-soaked sponge electrodes (50 × 70 mm) was positioned on the scalp at F3 and F4 locations (Figure 2) according to the 10–20 international system for electrode placement, determined using the Beam F3 location system [64]. Direct current stimulation was delivered using a DC-Stimulator Plus (Rogue Resolutions, Cardiff, UK) operated in accordance with published safety guidelines [65]. In the left tDCS condition, stimulation was anodal (excitatory) on F3 and cathodal (inhibitory) on F4. In the right tDCS condition, polarity was reversed (anode at F3 and cathode at F4). Current intensity was set at 1 milliamp, corresponding to a current density of 0.029 mA/cm^2^. The stimulation ascended during a 10-s ramp up to 1 mA, then stabilized within a 20 min plateau and faded out to 0 mA within 10 s. The plateau lasted only 15 s in the sham tDCS condition, then stimulation was discontinued after fading out. Participants were blind with respect to the type of stimulation. Blinding was ensured by the presence of two experimenters. One prepared the stimulation parameters whereas the second one who was interacting with the participant and in charge of the different steps of the experiment was blind to the stimulation parameters.

## 3. Results

### 3.1. Sleepiness and Vigilance

A repeated measure ANOVA conducted on subjective sleepiness (KSS) scores with within-subjects factor moment (KSS 1 (learning) vs. KSS 2 (RT1) vs. KSS 3 (RT2)) and between-subjects factor condition (TMR-anodal left tDCS, TMR-anodal right tDCS, TMR-sham tDCS, and TMR-only) failed to disclose significant differences in sleepiness scores from learning (3.42 ± 1.7) to retrieval (RT1 = 3.19 ± 1.1, RT2 = 3.45 ± 1.6, *F*(3, 65) = 0.48, *p* = 0.627). The main effect of condition and the condition by moment interaction were also non-significant (all *ps* > 0.370).

Similar analyses were conducted on two PVT parameters, i.e., the coefficient of variation and the reciprocal RT (mean 1/RT; [66]). Again, no differences were evidenced between the encoding and the recall session either using the coefficient of variation (PVT1 = 0.168 ± 0.08 vs. PVT2 = 0.159 ± 0.06, PVT3 = 0.162 ± 0.05, *F*(3, 65) = 0.81, *p* = 0.396) or the reciprocal RT (PVT1 = 0.0029 ± 0.0004 vs. PVT2 = 0.0030 ± 0.0005, PVT3 = 0.0030 ± 0.0003, *F*(3, 65) = 0.49, *p* = 0.488). The main effects of condition and the condition by moment interaction were also non-significant (all *ps* > 0.311).

Altogether, these results indicate no significant differences between the encoding and recall sessions in sleepiness and vigilance states, and that these states were not modulated by the intermediate administration of tDCS and TMR.

### 3.2. Pre-Stimulation Learning Session (IRT)

**Figure 3:**
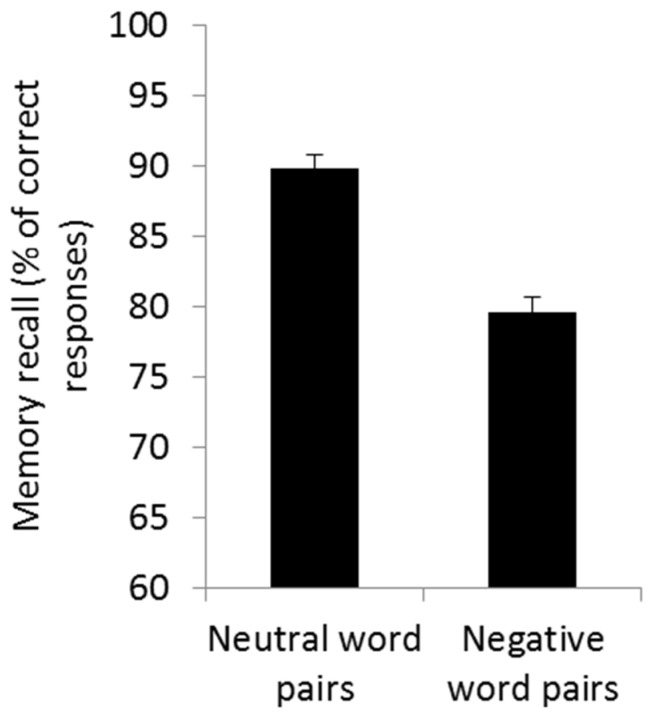
Memory performance for neutral and negative word pairs at the immediate recall task (IRT), expressed in percentage of correct responses. Error bars illustrate standard error.

A repeated measure ANOVA computed on the number of correctly retrieved word pairs at the end of learning (IRT) task with within-subject factors cueing (cued vs. non-cued) and emotion (neutral vs. negative) and between-subject factor condition (TMR-anodal left tDCS, TMR-anodal right tDCS, TMR-sham tDCS, and TMR-only) only disclosed a main effect of emotion (*F*(1, 65) = 35.16, *p* < 0.001, partial η^2^ = 0.351) with better learning for neutral (mean ± standard error 89.81 ± 0.98%) than negative word pairs (79.65 ± 1.1%; Figure 3), although performance was above the 75% cut-off score for both categories. No significant differences in retrieval score were observed between the word pairs to be subsequently cued and the word pairs not to be subsequently cued (*F*(1, 65) = 0.13, *p* = 0.717, partial η^2^ = 0.002), nor between the four conditions (*F*(3, 65) = 0.93, *p* = 0.433, partial η^2^ = 0.041). All other main and interaction effects were non-significant (all *ps* > 0.174, partial η^2^ < 0.054). Hence, pre-stimulation conditions were similar across the four experimental conditions.

### 3.3. Immediate Post-Stimulation Testing Session (RT1)

**Figure 4:**
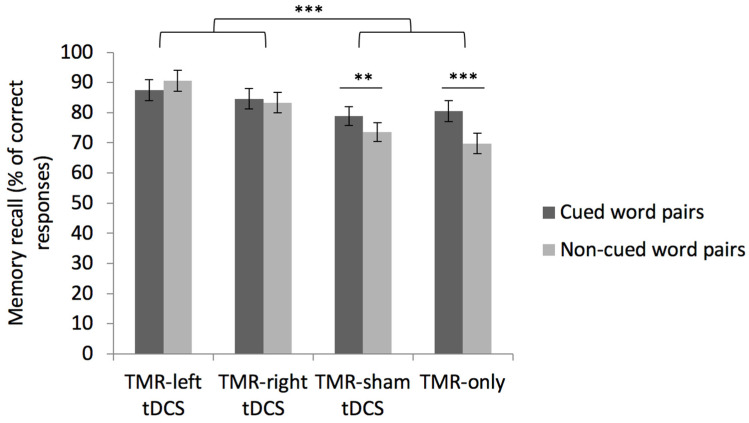
Memory performance for cued and non-cued word pairs at the first recall task (RT1) in the four conditions. Error bars illustrate standard error. ** *p* < 0.01, *** *p* < 0.001.

A repeated measure ANOVA computed on the percentage of correctly recalled word pairs with within-subject factors emotion (neutral vs. negative word pairs) and cueing (cued vs. non-cued) and between-subject factor condition (TMR-anodal left tDCS, TMR-anodal right tDCS, TMR-sham tDCS, and TMR-only) disclosed a main effect of condition (*F*(3, 65) = 4.58, *p* = 0.006, partial η^2^ = 0.174). There was a main effect of cueing (*F*(1, 65) = 6.78, *p* = 0.001, partial η^2^ = 0.094) with higher recall for cued (82.90 ± 1.7%) than non-cued (79.34 ± 1.7%) word pairs. In addition, there was a significant cueing by condition interaction (*F*(3, 65) = 4.41, *p* = 0.006; partial η^2^ = 0.169; see Figure 4). The cueing by emotion interaction effect was not significant (*F*(3, 65) = 0.69, *p* = 0.410, partial η^2^ = 0.010), and nor was the condition by cueing by emotion interaction effect (*F*(3, 65) = 0.64, *p* = 0.595, partial η^2^ =0.029). Similarly, the condition by emotion was not significant (*F*(1, 65) = 0.86, *p* = 0.467, partial η^2^ = 0.038).

Planned comparisons conducted on the significant between-subject factor condition showed that memory decline was significantly lower in conditions in which participants received real electrical stimulation (TMR-anodal left tDCS 88.82 ± 3.10% and TMR-anodal right tDCS 83.75 ± 3.05%) than in conditions in which no or a sham electrical stimulation was applied (TMR-sham tDCS 76.01 ± 2.93% and TMR-only 74.94 ± 3.10%; *F*(1, 65) = 12.58, *p* < 0.001, partial η^2^ = 0.157). A separate comparison between tDCS real (TMR-anodal left tDCS and TMR-anodal right tDCS) and sham (TMR-sham tDCS) conditions confirmed better performance in the tDCS real conditions (*F*(1, 51) = 8.89, *p* = 0.004, partial η^2^ = 0.148). The comparison between TMR-anodal left tDCS and TMR-anodal right tDCS conditions was non-significant (*F*(1, 31) = 1.86, *p* = 0.18; partial η^2^ = 0.057). Likewise, no significant differences were evidenced between the TMR-sham tDCS and the TMR-only conditions (*F*(1, 34) = 0.05, *p* = 0.82; partial η^2^ = 0.002).

Additional comparisons including the significant within-subject factor Cueing disclosed a significant Cueing effect in conditions in which no electrical stimulation was applied (TMR-sham tDCS, and TMR-only; *F*(1, 34) = 18.01, *p* < 0.001; partial η^2^ = 0.346), but not in conditions in which participants received real electrical stimulation (TMR-anodal left tDCS and TMR-anodal right tDCS; *F*(1, 31) = 0.21, *p* = 0.648, partial η^2^ = 0.007). A direct comparison between TMR-sham tDCS and TMR-only conditions indicates that the cueing effect was not significantly different (*F*(1, 34) = 2.11, *p* = 0.156, partial η^2^ = 0.058).

Altogether, these results suggest that tDCS significantly benefitted memory consolidation irrespective of the side of stimulation and of TMR effects. A benefit of targeted memory reactivation (i.e., cueing effect) was only observed in the TMR-sham tDCS, and TMR-only conditions. Finally, the emotional valence of the word pairs did not elicit any main or interaction effects, suggesting that neutral and negative memories equally benefitted from TMR, and that tDCS laterality did not interact with the emotional valence of the material.

### 3.4. Long Term Memory Consolidation (RT2)

**Figure 5:**
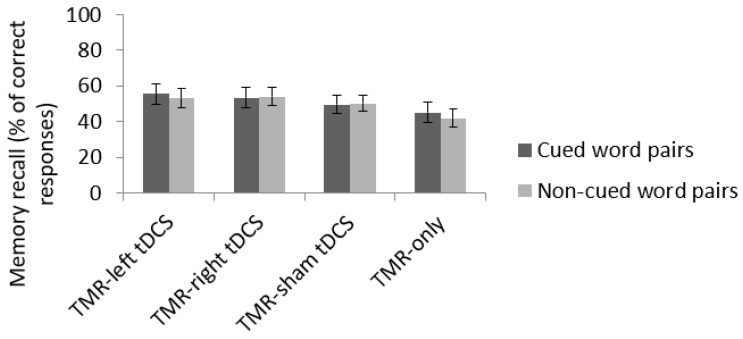
Memory forgetting for cued and non-cued word pairs from immediate (IRT) to delayed (RT2) recall in the four conditions. Error bars illustrate standard error.

Similarly, memory performance at delayed recall (one week later) was computed on the number of correctly retrieved learned word pairs expressed in percentage. The ANOVA with within-subject factors emotion (neutral vs. negative word pairs) and cueing (cued vs. non-cued) and between-subject factor condition (TMR-anodal left tDCS, TMR-anodal right tDCS, TMR-sham tDCS, and TMR-only) disclosed a main effect of emotion (*F*(1, 64) = 32.44, *p* < 0.001, partial η^2^ = 0.336) with better recall for neutral (mean ± standard error 56.31 ± 2.76%) than negative word pairs (44.43 ± 2.76%). All other main and interaction effects were non-significant (all *ps* > 0.185; all partial η^2^ < 0.072; Figure 5). Average forgetting across conditions was also significantly more pronounced in RT2 than RT1 (*F*(1, 64) = 95.371, *p* < 0.001, partial η^2^ = 0.598), and the interaction between conditions and testing session was non-significant (*F*(3, 64) = 0.245, *p* = 0.865, partial η^2^ = 0.011).

## 4. Discussion

In the present study, we first tested the hypothesis that providing auditory reminders (i.e., a TMR procedure) during a wakeful rest period would enhance memory consolidation for targeted items. Our results confirm a significant benefit of auditory reminders, but only in the TMR-only and TMR-sham tDCS conditions; that is in the absence of effective electrical stimulation. Secondly, we hypothesized that electrical stimulation of the DLPFC would improve the retention of word pairs and reinforce TMR effects. Our results partially fulfilled these predictions. Indeed, retrieval was significantly better in the TMR-anodal left and TMR-anodal right tDCS conditions than in the no- and sham stimulation conditions, confirming a beneficial effect of tDCS on memory consolidation, but this effect was generalized to all learned items irrespective of the TMR procedure, i.e., against the hypothesis that tDCS potentiates the selective enhancing benefits of TMR on memory. Finally, we tested the hypothesis that anodal excitatory stimulation of the right DLPFC associated with cathodal inhibitory stimulation of the right DLPFC would enhance the benefits of TMR for negative word pairs, eventually leading to greater benefits for negative than neutral cued word pairs. Results did not evidence polarity-dependent hemispheric effects on the consolidation of negative memories.

### 4.1. The Benefits of Auditory Cueing on Memory Consolidation

As stated above, there was a selective memory enhancement for cued as compared to non-cued word pairs in the TMR-only tDCS and TMR-sham tDCS conditions, while this effect was completely abolished in both TMR-anodal left tDCS and TMR-anodal right tDCS conditions. These results partially corroborate our primary hypothesis of a benefit of TMR for the consolidation of targeted items in memory. However, the fact that the cueing advantage was totally absent in both TMR-anodal left and TMR-anodal right tDCS conditions suggests that the selective effect of TMR was actually overshadowed by the global effect of tDCS. Indeed, no forgetting was observed in the recall task immediately after stimulation (RT1) for both cued and uncued items in both tDCS conditions, whereas forgetting was actually more pronounced for uncued than cued items in the TMR-only and TMR-sham tDCS conditions. Hence, the cueing benefit may have been abolished in effective tDCS conditions, possibly due to a better global memory retention masking the benefits of TMR. A limitation factor in the interpretation of these results is that although we ensured that the experimenter who was interacting with the participants was blind to the tDCS stimulation parameters (laterality and sham/actual), we did not debrief participants about their sensations and what they thought was their experimental condition.

It was shown already that different factors might determine the effectiveness of TMR. For instance, auditory cueing while awake was found to be mostly beneficial to rescue low reward value spatial stimuli from forgetting, while it did not impact high reward value stimuli [26]. In this latter study, low reward value stimuli actually also exhibited lower learning accuracy than high reward value items before the TMR intervention, suggesting that TMR while awake is mostly efficacious when learning levels are moderate. Similarly, a benefit of auditory cueing during sleep on memory for object locations was found only for items that were not already highly accurate before the intervention [67]. Hence, initially high encoding levels might explain why other studies failed to evidence even a moderate benefit of TMR while awake. Nevertheless, negative items were less efficiently learned than neutral ones in our present study, and still did not benefit more from TMR or tDCS, as there was no main or interaction effect of the emotional valence of the learned stimuli. Since both negative and neutral items were already learned above 75% accuracy in our study, it might be that this initial difference was insufficient to trigger differential effects either of TMR or tDCS.

### 4.2. The Effects of tDCS on Memory Consolidation

As expected, electrical stimulation of the DLPFC (TMR-anodal left and TMR-anodal right tDCS) led to significantly better recall performance as compared to the TMR-only and TMR-sham tDCS conditions. Therefore, 20 min of direct current stimulation over the left or right DLPFC during resting post-learning wakefulness can boost memory consolidation for declarative verbal material. These results are fitting with the report that tDCS over the left DLPFC while awake strengthens episodic memories and reduces further forgetting [43,44,68]. In particular, electrical stimulation of the DLPFC after encoding during undisrupted post-training wake was found beneficial for verbal episodic memory in older adults [43]. Inconsistently however, Kirov and colleagues [69] found that transcranial slow (0.75 Hz) oscillation stimulation (tSOS) applied 20 min after learning did not improve the retention of declarative memories, although it increased endogenous EEG slow oscillation as well as theta activity. However, methodological differences (electrode size and montage) and study design (post-learning electrical stimulation delayed by 20 min) might explain these discrepancies. In our study, the reactivation of memories triggered by the TMR procedure might have contributed to the memory benefits following tDCS. Because the TMR procedure reinstated the activation of the mnemonic traces, the electrical brain stimulation might have stabilized memory through a process of consolidation or reconsolidation. Accordingly, Javadi and Cheng [70] found that anodal stimulation of the left DLPFC during a consolidation interval improved memory performance, but only when the memory traces were reactivated during the stimulation using an old-new recognition task. Together, these results point out the combination of memory reactivation procedures and electrical brain stimulation as a promising method to enhance memory consolidation.

The exact mechanisms by which tDCS improves memory consolidation when applied during a resting state following learning are still unclear, even if we have increasing knowledge about the effects of tDCS on cortical excitability [71,72]. Neurophysiological studies showed that anodal tDCS induces cortical excitability and enhances NMDA receptor plasticity [71,73,74,75]. Memory formation is also known to depend on changes in synaptic strength such as synaptic tagging and long-term potentiation [76]. Thus, the benefits of tDCS over the DLPFC might primarily stem from a direct modulation of synaptic plasticity processes within prefrontal areas, in itself favoring memory reorganization, eventually leading to improved recall accuracy. Secondly, as mentioned above, memory consolidation processes are thought to rely on the offline reactivation of learning-related neural activity [3,4,5]. The reactivation of declarative, hippocampus-dependent memories through a dialogue between hippocampal and neocortical areas is possibly mediated by slow oscillatory activity while awake [23] like during sleep [2]. Transcranial DCS-related increased excitability in prefrontal regions could also favor connections with remote memory-related areas. For instance, anodal tDCS over the primary motor cortex was shown to efficiently increase functional coupling with subcortical thalamus regions [77]. Likewise, theta frequency-modulated oscillatory anodal tDCS over the left posterior parietal cortex before learning [42] improved subsequent performance. In the present case, it can be speculated that increasing brain excitability within prefrontal areas might boost connections with hippocampal areas, thereby increasing the susceptibility of memory traces to be reactivated. However, we cannot confirm the specific involvement of the DLPFC as our study did not include a control condition with the stimulation of a region not supposed to be involved in these processes, such as the primary motor cortex.

Hemispheric specialization of the DLPFC in learning and memory is a matter of debate in the literature. It has been proposed that the left DLPFC mostly supports encoding while the right DLPFC mostly supports retrieval [78], or that the left DLPFC supports the consolidation of verbal material while the right DLPFC processes non-verbal material [79]. Although our results support the idea that tDCS over the DLPFC during a consolidation episode benefits memory for verbal declarative material, we did not find an effect of tDCS polarity on behavioral outcomes. As well, prior studies led to contrasting results regarding an asymmetric involvement of the DLPFC. As stated above, Sandrini et al. [68] showed that 15 min of tDCS over the left DLPFC improves recall for verbal material. Several studies also support the proposal of a specialization of the left DLPFC in memory consolidation [37,38,68,70]. Nonetheless, an implication of the right DLPFC in the reactivation and consolidation of episodic memories was evidenced in a fMRI study [29] with increased activity in the right lateral prefrontal cortex after re-exposure to an odor associated with the context of learning. Likewise, 1 Hz repetitive transcranial magnetic stimulation (rTMS) applied after memory reactivation over the same region was found to improve retention of episodic memories [80].

### 4.3. Consolidation of Emotional Memories and Lateralisation of tDCS Polarity

We hypothesized that anodal excitatory stimulation of the right DLPFC (with cathodal inhibitory stimulation of the left DLPFC) would increase the selective enhancement effect of TMR for negative word pairs. However, our results did not confirm a polarity-dependent effect of tDCS for the consolidation of negative items. Therefore, we cannot conclude that the polarity of tDCS applied during a consolidation interval modulates TMR for negative memories. Like for a global TMR effect however, it is possible that the main and powerful effect of tDCS on overall memory performance masked the specific effect of tDCS polarity on the consolidation of negative word pairs.

Although this possibility should be investigated in further studies, others similarly failed to evidence inter-hemispheric dissociations between left and right DLPFC in the processing of negative emotions, or even obtained unexpected opposite effects. For instance, anodal tDCS over the left DLPFC was found to facilitate the recognition of negative and positive facial expressions (with a more pronounced benefit for positive emotions), while it did not impact the emotional state of the participants [81]. Similarly, Penolazzi et al. [82] found that right anodal/left cathodal stimulation over fronto-temporal regions facilitated recall for pleasant images, whereas left anodal/right cathodal stimulation improved recall for unpleasant images. Notwithstanding, our results should be taken cautiously as neutral word pairs were better encoded than negative ones at the end of learning already. Superior learning for neutral items is surprising given that arousing negative emotional material is usually better encoded [83]. Indeed, most studies having investigated the influence of emotional valence in the time course of memory consolidation found emotional memories to be usually better remembered over time [84], probably because the amygdala mediates the organization of memories in the hippocampus and the neocortex [85,86].

In the present study, participants were asked to learn word pairs while listening to an emotionally congruent sound. They were not asked to learn the sound. Therefore, the sounds acted as contextual (specific to the word pair) cues rather than elements to be learned. The benefits of TMR on memory recall were expected to be related to the replay of the auditory cues. We speculate here that the weaker encoding of negative associations is related to the fact that the arousing negative sounds acted as contextual cues. Indeed, a highly arousing emotional context can impair memory formation processes. For instance, a fMRI study [87] highlighted negative correlations between recall performance and amygdala activation during the encoding of negative word and neutral face pairs, suggesting that amygdala activation induced by negative emotions may disrupt associative memory performance. Furthermore, Zhang et al. [88] showed that highly arousing emotional pictures impaired the recognition of neutral words. They found that highly arousing contexts elicited more positives ERPs (as compared to low arousing contexts), suggesting their automatic attentional capture and the presence of cognitive resources engaged to overcome the interference induced by high-arousing context, that would eventually impair learning. Thus, it cannot be excluded that the arousing effect of the negative sounds associated with the word pairs was too high, which would have shifted the participants’ attention toward the sounds, to the detriment of the associated negative word pairs as compared to the neutral word pairs. Prior studies also found a specific deleterious effect of arousing negative emotions on associative learning [89,90,91]. Such attentional shift might have interfered with encoding and later retrieval processes, possibly masking a specific effect of right anodal/left cathodal stimulation of the DLPFC.

In addition to an interference effect due to a high-arousing context, another possible and complementary explanation for a weaker encoding of negative associations relies on the differential effects of negative emotion on item versus associative memory. Indeed, it is commonly assumed that negative emotional content enhances memory for the content by boosting amygdala activity, while the binding of items and context subtended by the hippocampal activity might be impaired, resulting in a detrimental effect of negative emotions on associative memory. Guez et al. [90] found that negative emotional arousal had a more pronounced deleterious effect on associative memory in comparison to item memory. Similarly, Bisby et al. [91] found a beneficial effect of negative emotion on item memory, whereas it impaired associative memory. Therefore, another explanation for a weaker encoding of negative associative word pairs observed in our study might stem from a dual memory process that differentially triggers an emotional advantage for item memory but a disadvantage for associative memory.

### 4.4. No Long-Term Benefits of TMR and tDCS

A 30 to 40% forgetting rate was similarly observed in all conditions when retested one week later, suggesting that awake TMR and tDCS-related benefits on memory consolidation are short lived, contrary to prior reports (e.g., [43,44,45]). It is possible that the high level of memory performance achieved at the first recall session may have masked the long-term benefit of TMR and tDCS in our study. For instance, in the Flöel et al. study [45], elderly subject might have had more room for memory improvement, increasing the potential tDCS-related memory improvement in the long-term. Another possible explanation is that presenting auditory reminders during a period of wakeful rest initially boosts the associated memories but concomitantly puts those into a more labile condition more susceptible to external interference, eventually leading to forgetting. For instance, re-exposure to a contextual odor while awake was shown to impair the retrieval of image location [29], probably because memories are more labile after reactivation and need to be reconsolidated [70,92], but see [93]. Additionally, the time interval between learning and delayed recall was seven days without any reminders. It is possible that cueing memories over consecutive days might have led to identifiable benefits in the long-term. Further studies are needed to disentangle the temporal effects of both tDCS and TMR techniques.

## 5. Conclusions

In summary, we have shown in the present study that TMR during a wakeful resting period benefits short-term memory consolidation. However, concomitant tDCS either on right or left DLPFC gave rise to much higher but unspecific memory enhancements, hence abolishing or at least overshadowing the TMR advantage. Finally, our results did not evidence a polarity-dependent hemispheric effect of tDCS on the consolidation of emotional negative memories. Noticeably, stimulation of the DLPFC during a 20 min period following learning was found to be beneficial for the consolidation of verbal declarative memories. By increasing cortical excitability in prefrontal areas, tDCS might favor the hippocampo-cortical dialogue subtending memory consolidation processes.

## Data Availability

Raw data are publicly available at https://osf.io/4a3d5/ (accessed on 27 May 2020).
